# Associations Between Microbial Depletion and Autonomic Dysregulation in Binge‐Eating Disorder

**DOI:** 10.1002/eat.70139

**Published:** 2026-05-31

**Authors:** Shuang Liang, Esben Strodl, Leanne Barron, Matthew Bambling, Luis Vitetta

**Affiliations:** ^1^ School of Psychology and Counselling, Faculty of Health, QUT Brisbane Queensland Australia; ^2^ QUT Eating Disorder Clinic Brisbane Queensland Australia; ^3^ Faculty of Medicine University of Queensland Brisbane Queensland Australia; ^4^ Faculty of Medicine and Health University of Sydney Sydney New South Wales Australia

**Keywords:** autonomic nervous system, binge‐eating disorder, dysbiosis, gut microbiome, heart rate variability, major depressive disorder, microbiota‐gut‐brain axis, *Pediococcus*, vagus nerve

## Abstract

**Objective:**

The interplay between the gut microbiome and autonomic nervous system remains unexplored in binge‐eating disorder (BED). We aimed to explore specific microbial alterations in BED and examine their potential association with cardiac vagal tone as a distinct bio‐behavioral phenotype.

**Method:**

Women with BED and co‐occurring major depressive disorder (BED+MDD; *n* = 19) were compared to strictly matched controls with MDD alone (*n* = 38) to isolate BED‐specific effects. We analyzed the gut microbiome via 16S rRNA sequencing and assessed cardiac vagal tone using short‐term heart rate variability (specifically normalized high‐frequency power, HFnu). Nutritional intake was analyzed to explore diet‐microbiome interactions.

**Results:**

While global diversity did not differ between groups, differential abundance analysis identified lower relative abundance of several fermentative taxa, including *Catenibacterium*, *Acidaminococcus*, *Pediococcus*, *Fusobacterium*, *Megasphaera*, and *Prevotella* in the BED group. Notably, a cross‐system association emerged exclusively in the BED group: the depletion of *Pediococcus* was strongly correlated with reduced vagal tone (HFnu; *p* = 0.003) and specific micronutrient patterns. This relationship was absent in the MDD‐only controls.

**Discussion:**

Our findings demonstrate a BED‐specific link between the depletion of the fermentative guild, autonomic instability, and dietary energy intake. This linked state may reflect a physiological response to the acute substrate surges characteristic of binge‐eating, potentially compromising gut‐brain axis homeostasis. Future research incorporating direct measures of binge behavior and luminal environment is required to validate whether these microbial patterns represent a causal mechanism or a reproducible physiological marker of the disorder.

## Summary

- *Specific microbial alterations:* Unlike depression alone, binge‐eating disorder (BED) is characterized by a synchronized depletion of fermentative taxa, specifically *Pediococcus* and *Megasphaera*.
- *Gut‐brain associations:* A distinct cross‐system association was observed in BED, where the depletion of *Pediococcus* was significantly correlated with reduced cardiac vagal tone (HRV), a relationship absent in matched MDD controls.
- *Mechanistic implication:* Results suggest a bio‐behavioral feedback loop where binge‐induced gut instability is associated with alterations in the short‐chain fatty acid (SCFA)‐vagus axis, highlighting a novel microbial‐neural pathway in BED pathophysiology.

## 1 Introduction

Binge‐eating disorder (BED) is characterized not only by recurrent episodes of loss of control overeating (American Psychiatric Association 2013) but also by profound dysregulation in physiological stress systems (American Psychiatric Association 2013; Mathes et al. 2009). While the gut‐brain axis has emerged as a key regulator of eating behavior and emotional health (Cryan et al. 2019), current understanding of BED pathophysiology remains fragmented. Most microbiome studies have focused on moderate to severe gut dysbiosis associated with obesity (Giel et al. 2022), involving reduced diversity and pathogenic overgrowth ranging from minor shifts to chronic dysregulation (DeGruttola et al. 2016). However, research has largely overlooked how the specific physiological context of BED might relate to specific microbial alterations (Giel et al. 2022). Furthermore, although the vagus nerve is the primary conduit for bidirectional gut‐brain communication, few studies have simultaneously examined microbial composition and autonomic function in this population.

The gastrointestinal environment in BED is uniquely challenging. Repeated cycles of restriction and binge eating impose acute fluctuations in nutrient availability, osmotic load, and luminal pH, creating a volatile ecosystem that may selectively deplete metabolically specialized taxa (Flint et al. 2012; Moraes et al. 2023; Zipfel et al. 2006). Among these, members of the *Lactobacillaceae* family are of particular interest due to their dependence on specific micronutrients (including vitamins, amino acids, and carbohydrates) (Lv et al. 2017) for growth, as well as evidence that certain *Lactobacillus* strains may influence host vagal signaling in experimental models (Bravo et al. 2011; LeBlanc et al. 2013). These taxa are often metabolically fastidious, requiring stable nutrient availability for persistence. Accordingly, binge/restrict cycles characteristic of BED marked by irregular nutrient intake, low micronutrient density foods, and rapid fluctuations in substrate availability (Lotfi Yagin et al. 2024) may destabilize these taxa and alter microbial ecology. It remains unclear, however, whether the depletion of such psychobiotics is merely a bystander effect of poor diet or a mechanistic link contributing to the sustained autonomic dysregulation observed in individuals with BED (Ávila et al. 2025; Godfrey et al. 2019).

To address this gap, the present study adopted a multimodal approach integrating dietary analysis, gut microbiome analysis, psychological symptom assessment, and heart rate variability to capture the full bio‐behavioral phenotype. Crucially, we employed a cross‐sectional sample design by comparing individuals with comorbid BED and major depressive disorder (MDD) against an MDD‐only control group. Given the high prevalence of this comorbidity (Kowalewska et al. 2024), this design allows us to isolate physiological alterations specific to the binge‐eating pathology, controlling for the confounding effects of depression itself. We assessed whether, among individuals with BED, nutritional inadequacies relate to the depletion of specific vagal‐modulating bacteria, which in turn correlates with reduced heart rate variability.

## 2 Method

This study was a secondary analysis of baseline data from a 16‐week, double‐blind, randomized, placebo‐controlled clinical trial (Strodl et al. 2024). The parent trial was prospectively registered with the Australian and New Zealand Clinical Trials Registry (ACTRN12617000419369), received ethics approval from both the University of Queensland and the Queensland University of Technology, and was conducted in accordance with CONSORT guidelines as described previously (Strodl et al. 2024). Data was collected from the participants at the Queensland University of Technology's Kelvin Grove Campus in Brisbane Australia. Data collection began July2018 and ended in May 2020.

Participants were adults aged 18 years and older with a current diagnosis of MDD and comorbid diagnoses that were confirmed by a clinical psychologist using the Structured Clinical Interview for DSM‐5 Research Version (SCID‐5‐RV) (First et al. 2015). From the parent RCT cohort, all available participants meeting DSM‐5 criteria for BED with comorbid MDD (*n* = 19) at baseline (*n* = 19) were identified as the case group. A comparison group with MDD without any eating disorder diagnoses (*n* = 38) was drawn from the same baseline cohort and matched to the BED group based on age, sex, and body mass index (BMI) at a ratio of 2:1 in accordance with standard published guidelines (Schulz and Grimes 2002). However, data on race and ethnicity were not available because these variables were not collected in the original source database used for this secondary analysis.

### 2.1 Psychological, Dietary, Physiological, and Microbiome Measures

Depressive symptoms were assessed using the Beck Depression Inventory–II (Beck et al. 1996), anxiety symptoms using the Generalized Anxiety Disorder Scale (Spitzer et al. 2006), and perceived stress using the 10‐item perceived stress scale (Cohen et al. 1983).

Dietary intake was assessed at baseline using self‐reported three‐day food records (2 weekdays and 1 weekend day). Records were analyzed using FoodWorks 10 Professional (Xyris Software, Brisbane, Australia), which draws on the Australian Food and Nutrient Database (AUSNUT) to estimate total energy intake, macronutrient intake, micronutrient intake, and food group consumption.

Cardiac autonomic activity was assessed through short‐term resting‐state Heart Rate Variability (HRV) recordings of at least 5 min, in accordance with established standards for frequency‐domain analysis. Raw data were acquired and processed using LabChart 8 software (Version 8.x, AD Instruments, Australia) (ADInstruments 2024). Normalized High‐Frequency Power (HFnu) was calculated as the high‐frequency power divided by the total power minus very‐low‐frequency power and was selected as the primary index of cardiac vagal control, or parasympathetic nervous system tone, to minimize the confounding impact of inter‐individual variations in total power (Laborde et al. 2017; Pham et al. 2021).

Baseline gut microbiome composition was determined using sequencing‐based methods. Samples were processed using 16S rRNA gene sequencing targeting the V3 and V4 regions, sequenced on the Illumina MiSeq platform, and analyzed using MiSeq Reporter software (MSR) with the Illumina Metagenomics workflow (Ritchie et al. 2023). These details are provided to ensure transparency regarding taxonomic resolution and detection bias.

### 2.2 Statistical Analysis

All statistical analyses were conducted in R (version 4.4.2). Alpha diversity (observed genus richness and Shannon index) and beta diversity (Bray–Curtis dissimilarity and visualized by principal coordinates analysis [PCoA]) were calculated at the genus, family, and order levels. Group differences in alpha diversity were examined using Wilcoxon rank‐sum tests, while between‐group differences in community structure were assessed using permutational multivariate analysis of variance (PERMANOVA).

Differential abundance analyses at the genus, family, and order levels were performed using DESeq2 (Love et al. 2014). Taxa identified as differentially abundant between BED and control groups were selected as candidate taxa for subsequent analyses. Taxa were identified as differentially abundant using a Benjamini–Hochberg false discovery rate (FDR) threshold of 0.05 and were selected as candidates for subsequent analyses.

Nutritional analysis encompassed macronutrients, micronutrients, and food groups. After excluding participants with biologically implausible dietary entries, the remaining data were compared between groups using independent *t*‐tests or Mann–Whitney *U* tests, depending on data distribution. Cliff's delta was additionally calculated as effect size estimate for between‐group differences. Exploratory Spearman correlation analyses were conducted to examine associations between candidate taxa and psychological measures, HRV indices, and dietary variables. Analyses were performed for the full sample and separately within the BED and control groups. To mitigate the leverage of extreme outliers and facilitate clear graphical representation, skewed dietary variables were log‐transformed prior to visualization, noting that Spearman's rho remains invariant to such monotonic transformations. These analyses were conceptually guided by the proposed relationship between dietary inadequacies, microbial depletion, and reduced HRV in BED. Given the exploratory nature of the correlation analyses and the large number of variables examined, results were primarily interpreted based on unadjusted *p* values, with Benjamini–Hochberg FDR–adjusted values reported for transparency.

To ensure the robustness of the findings, we calculated the prevalence of key taxa. Furthermore, potential confounding was addressed using partial correlation models adjusting for both BDI and total energy intake.

## 3 Results

### 3.1 Participant Characteristics and Clinical Profiles

A total of 57 participants were included in the study, consisting of 19 individuals with BED and 38 age‐ and BMI‐matched controls. The two groups were well‐matched across all demographic, lifestyle, and clinical variables, including age, BMI, and medication use (Table 1). Regarding clinical profiles, while the BED group showed a nonsignificant trend toward higher depression scores, no statistically significant differences were observed between groups in anxiety (GAD), perceived stress (PSS), and our HRV index (HFnu). Depression severity (BDI scores) was included as a covariate to ensure that the observed associations were specific to the BED phenotype rather than a reflection of heightened affective burden. Additionally, total energy intake was adjusted to isolate the independent effects of specific micronutrients from the overall caloric load, thereby addressing the high collinearity between individual nutrient intake and the magnitude of binge‐eating episodes (Willett and Stampfer 1986). This high degree of homogeneity between groups effectively minimizes the impact of common confounding factors on the subsequent gut microbiota analyses.

**TABLE 1:** Demographics and clinical characteristics.

Characteristic	Overall*N* = 57^a^	Control*N* = 38^a^	BED*N* = 19^a^	*p* ^b^
Age (years)	37 (13)	36 (12)	40 (14)	0.37
BMI (kg/m^2^)	32 (8)	32 (8)	32 (8)	0.95
Gender				> 0.99
Male	12 (21%)	8 (21%)	4 (21%)	
Female	43 (75%)	29 (76%)	14 (74%)	
Other	2 (3.5%)	1 (2.6%)	1 (5.3%)	
Relationship status				0.81
Married	19 (33%)	12 (32%)	7 (37%)	
De facto	8 (14%)	6 (16%)	2 (11%)	
Divorced/separated	5 (8.8%)	3 (7.9%)	2 (11%)	
Dating	5 (8.8%)	4 (11%)	1 (5.3%)	
Single	19 (33%)	13 (34%)	6 (32%)	
Other	1 (1.8%)	0 (0%)	1 (5.3%)	
Antibiotics use (Past 12 months)^d^	32 (56%)	20 (53%)	12 (63%)	0.45
Antidepressant medication				0.85
None	34 (60%)	23 (61%)	11 (58%)	
SSRI/SNRI	23 (40%)	15 (39%)	8 (42%)	
Alcohol consumption (drinks/day)	0.64 (1.21)	0.64 (1.31)	0.64 (1.01)	0.97
History of Smoking	13 (23%)	8 (21%)	5 (26%)	0.74
Depression (BDI score)	30 (11)	28 (11)	34 (10)	0.06
Anxiety (GAD score)	10.5 (5.2)	9.8 (5.4)	12.0 (4.6)	0.16
Perceived stress (PSS score)	26.6 (5.2)	26.5 (5.3)	26.7 (5.3)	0.98
HRV high frequency (n.u.)^c^	39 (18)	38 (19)	41 (16)	0.53
HRV LF/HF ratio^c^	2.46 (2.89)	2.85 (3.60)	2.01 (1.76)	0.49
Comorbid GAD	33 (57.9)	23 (60.5)	10 (52.6)	0.77
Comorbid PDD	19 (33.3)	13 (34.2)	6 (31.6)	1.00

*Note:* Data presented as mean (SD) or *n* (%).

Abbreviations: BDI, beck depression inventory; BED, binge‐eating disorder; BMI, body mass index; GAD, generalized anxiety disorder; HRV, heart rate variability; LF/HF, low frequency/high frequency; PDD, persistent depressive disorder; PSS, perceived stress scale; SD, standard deviation; SNRI, Serotonin–Norepinephrine Reuptake Inhibitor; SSRI, selective serotonin reuptake inhibitor.

^a^ Mean (SD); *n* (%).

^b^ Wilcoxon rank‐sum test; Wilcoxon rank‐sum exact test; Fisher's exact test; Pearson's Chi‐squared test.

^c^ Data were unavailable for some participants (Group BED: *n* = 1; Group Control: *n* = 17; Total: *n* = 18).

^d^ Antibiotics use excludes the 4 weeks prior to sampling.

### 3.2 Global Microbiota Structure (Alpha and Beta Diversity)

**FIGURE 1:**
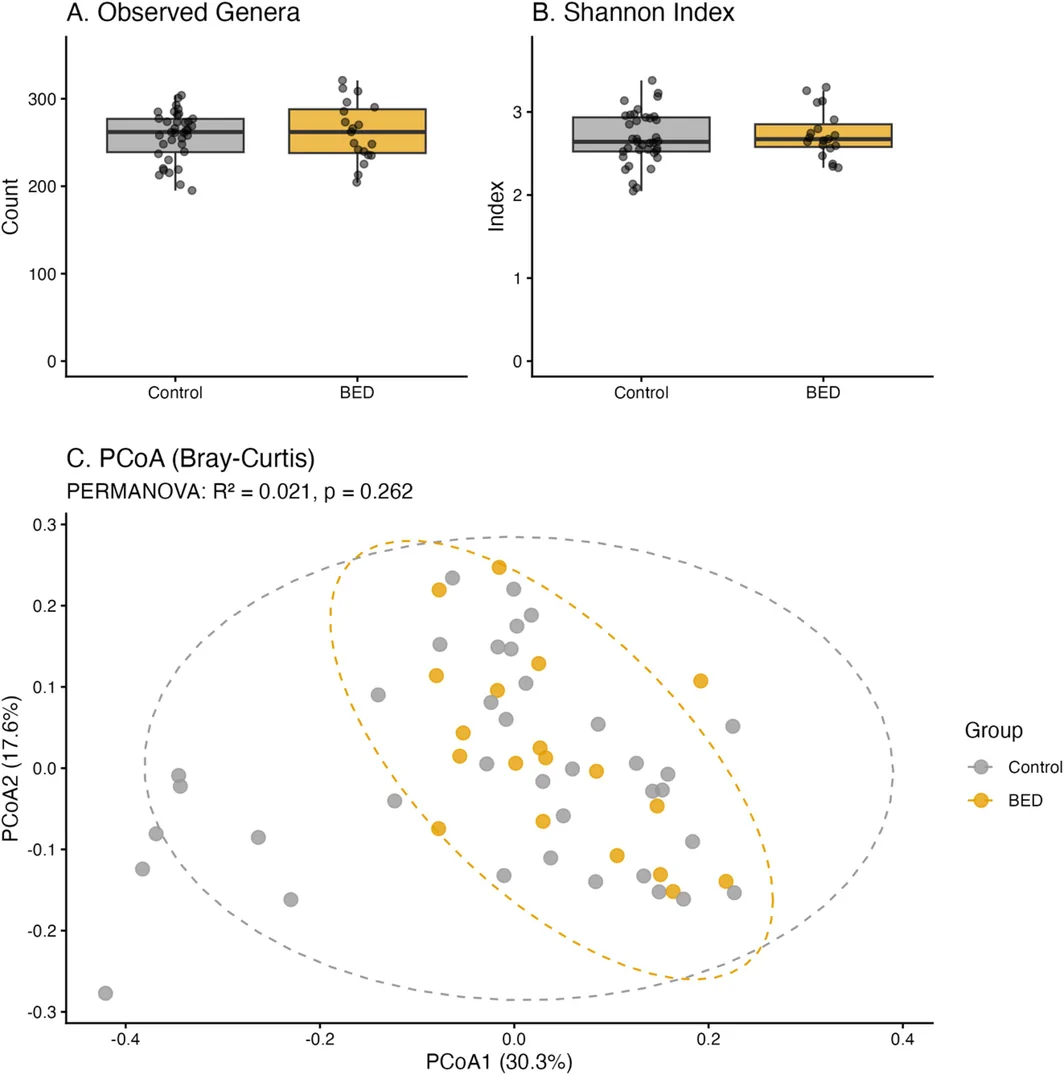
Alpha and beta diversity of gut microbiota in BED and control groups. (A and B) Alpha diversity indices including Observed Species and Shannon Index compared using the Wilcoxon rank‐sum test. Boxes represent the interquartile range (IQR) with the median indicated by the center line. (C) Principal coordinates analysis (PCoA) plot based on Bray‐Curtis distance, with ellipses representing 95% confidence intervals for each group. Statistical significance of community composition was assessed by PERMANOVA. BED, binge‐eating disorder and major depressive disorder; Control, major depressive disorder. *p* < 0.05.

No significant differences were observed in alpha diversity (Observed richness, Shannon index) or beta diversity (Bray–Curtis dissimilarity) between BED and control groups at the genus, family, or order levels (all *p* > 0.05; Figure 1), indicating that BED is not characterized by large‐scale shifts in global microbial community structure in the gut.

### 3.3 Differential Abundance Across Taxonomic Levels

**FIGURE 2:**
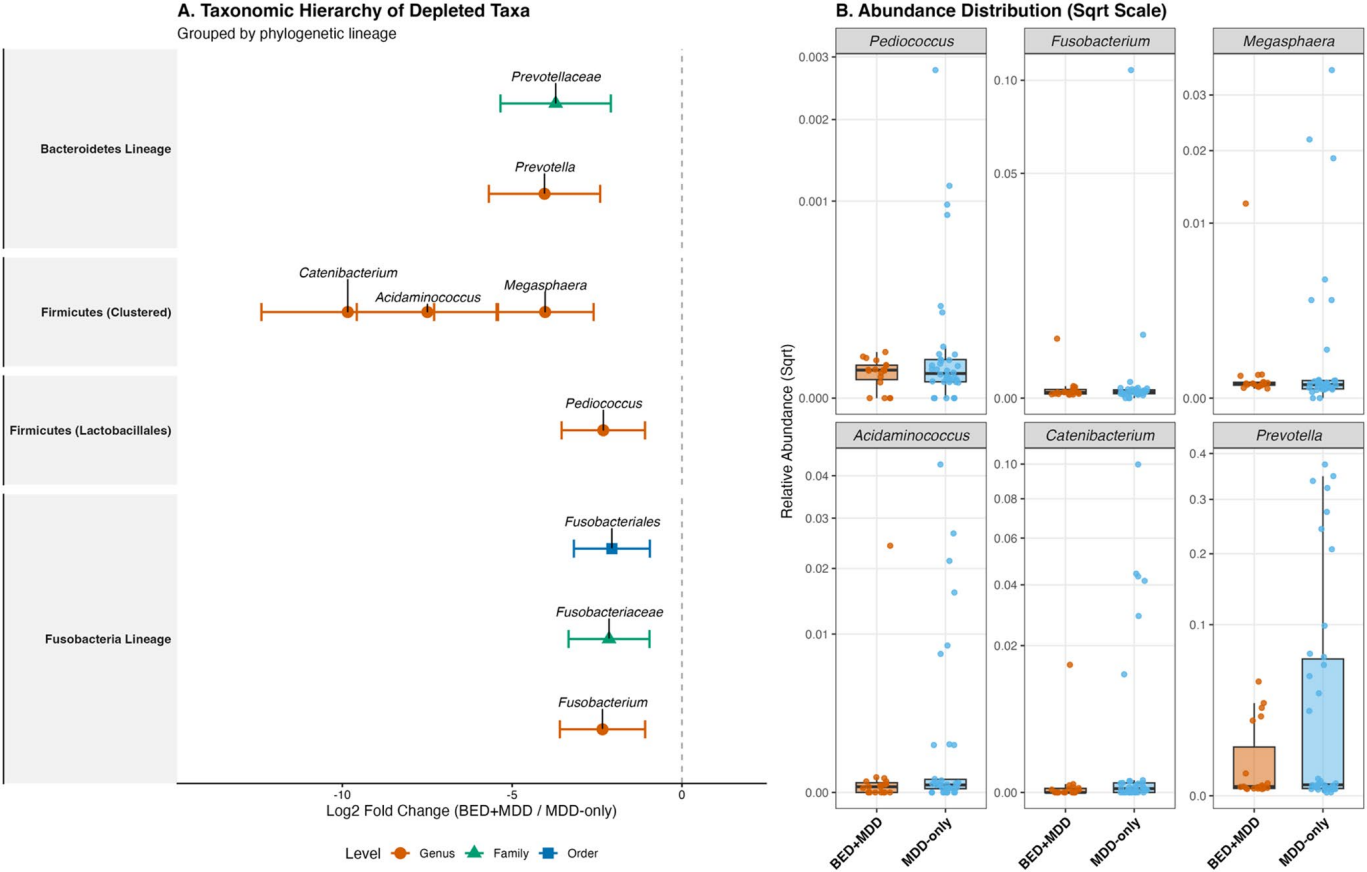
Phylogenetic profiling and abundance distribution of taxa depleted in the BED+MDD phenotype. *Note*. (A) Forest plot displaying taxa significantly depleted in the BED+MDD group identified via DESeq2 analysis (false discovery rate [FDR] adjusted *p* < 0.05). Points represent Log_2_ fold change, and horizontal error bars indicate 95% confidence intervals. Taxa are color‐coded and shaped by their respective taxonomic levels (Order, Family, and Genus). Negative values on the *x*‐axis represent lower abundance in the BED+MDD group relative to MDD‐only group. (B) Boxplots illustrating the distribution of relative abundance for six key genera identified in the discovery phase. The y‐axis is square‐root transformed to visualize differences in low‐abundance taxa effectively. Individual data points (colored dots) are overlaid to demonstrate sample variability. Orange indicates the BED+MDD group (*n* = 19), and blue indicates the MDD‐only group (*n* = 37). BED, binge‐eating disorder; MDD, major depressive disorder.

To identify specific microbial alterations associated with BED, we performed differential abundance analysis using DESeq2. As shown in Figure 2, significant depletions were observed across multiple taxonomic levels.

At the higher taxonomic levels, the order *Fusobacteriales* (Log_2FC = −2.06) and its family *Fusobacteriaceae* (Log_2FC = −2.15) were significantly reduced in the BED group (both FDR < 0.05), indicating a robust lineage‐wide alteration.

At the genus level, six genera were significantly depleted. The most profound reductions were found in *Catenibacterium* (Log_2FC = −9.84) and *Acidaminococcus* (Log_2FC = −7.49). Additionally, *Pediococcus* (Log_2FC = −2.32) and *Fusobacterium* (Log_2FC = −2.34) showed significant reductions with 95% confidence intervals excluding zero (Table S1). These findings highlight that while some alterations (e.g., *Fusobacterium*) reflect broader taxonomic shifts, others (e.g., *Pediococcus*) represent specific genus‐level alterations in BED pathology.

### 3.4 Disease‐Specific Associations Between Microbiota and Dietary Pattern

Consistent with prior observations that standard dietary recalls primarily capture habitual intake in BED populations (Bartholome et al. 2013; Wehling and Lusher 2019), no significant differences in macronutrients, food groups, and micronutrient intake were observed between BED and MDD groups (Table 2), This suggests that the food records likely reflect habitual intake rather than episodic binge consumption. Importantly, outlier detection analysis confirmed no extreme outliers in total energy intake within the BED group, ensuring the stability and representativeness of the dietary data distribution.

**TABLE 2:** Comparison of dietary intake between BED and MDD groups.

Variable	Control (*n* = 35)	BED (*n* = 19)	*p*	Cliff's delta
Part A: Macronutrients				
Energy (kJ)	8946 ± 2879	10,517 ± 5725	0.23	0.20
Protein (g)	92.8 ± 35.3	106.5 ± 39.8	0.18	0.22
Total fat (g)	96.7 ± 46.9	101.5 ± 72.5	0.91	−0.02
Carbohydrates (g)	204 ± 65	260 ± 158	0.18	0.23
Fiber (g)	20.3 ± 8.0	24.6 ± 14.1	0.26	0.19
Sugars (g)	90 ± 36	131 ± 147	0.42	0.14
Starch (g)	112.1 ± 39.3	125.5 ± 53.6	0.35	0.12
Part B: Micronutrients				
Folate (μg)	437 ± 168	530 ± 217	0.12	0.26
Magnesium (mg)	326 ± 109	404 ± 160	0.70	0.30
Zinc (mg)	10.5 ± 3.5	11.9 ± 4.9	0.30	0.13
Calcium (mg)	867 ± 366	1118 ± 643	0.19	0.22
Thiamin (mg)	1.54 ± 1.00	1.60 ± 0.66	0.29	0.18
Riboflavin (mg)	1.94 ± 0.96	2.16 ± 1.02	0.28	0.18
Niacin (mg)	24.7 ± 12.3	28.8 ± 10.9	0.08	0.29
B6 (mg)	1.88 ± 1.95	1.71 ± 0.72	0.21	0.21
B12 (μg)	4.36 ± 1.93	4.68 ± 2.03	0.70	0.06
Vitamin C (mg)	91.4 ± 73.4	101.8 ± 67.5	0.56	0.10
Vitamin E (mg)	12.20 ± 5.78	12.59 ± 6.70	0.86	0.03
Retinol (μg)	343 ± 210	394 ± 253	0.48	0.12
Betacarotene (μg)	3326 ± 3522	2954 ± 2732	0.97	−0.01
Sodium (mg)	2707 ± 930	2361 ± 746	0.14	−0.17
Potassium (mg)	2967 ± 926	3603 ± 1423	0.13	0.25
Phosphorus (mg)	1483 ± 380	1784 ± 748	0.16	0.23
Iron (mg)	11.65 ± 5.55	12.31 ± 6.12	0.83	0.04
Selenium (μg)	77.9 ± 34.3	91.0 ± 37.7	0.16	0.24
Iodine (μg)	158.7 ± 58.8	210.7 ± 88.4	0.07	0.30
Part C: Food groups				
Grains (serve)	4.95 ± 2.04	5.26 ± 2.89	0.68	0.05
Fruit (serve)	0.91 ± 0.87	1.02 ± 0.92	0.73	0.06
Vegetables (serve)	3.60 ± 2.49	4.22 ± 4.17	0.96	−0.01
Protein Foods (serve)	2.53 ± 1.43	3.04 ± 2.56	0.75	0.06
Dairy (serve)	1.65 ± 0.95	2.57 ± 2.07	0.10	0.28
Oil equivalents (tsp)	7.84 ± 4.41	7.72 ± 3.71	0.59	0.09
Added Sugars (tsp)	9.9 ± 7.9	16.2 ± 32.2	0.72	−0.06
Alcoholic drinks (sd)	0.71 ± 1.42	0.97 ± 1.78	0.51	0.10

*Note:* Data are presented as Mean ± Standard Deviation (SD). Dietary intake represents absolute daily amounts without energy density adjustment. Three extreme outliers in the control group were excluded from this analysis due to biologically impossible data entry errors. Statistical differences between groups were analyzed using Welch's *t*‐test for normally distributed variables and the Wilcoxon rank‐sum test for non‐normally distributed variables. Cliff's delta was calculated to estimate effect size.

Abbreviation: BED, binge‐eating disorder.

**FIGURE 3:**
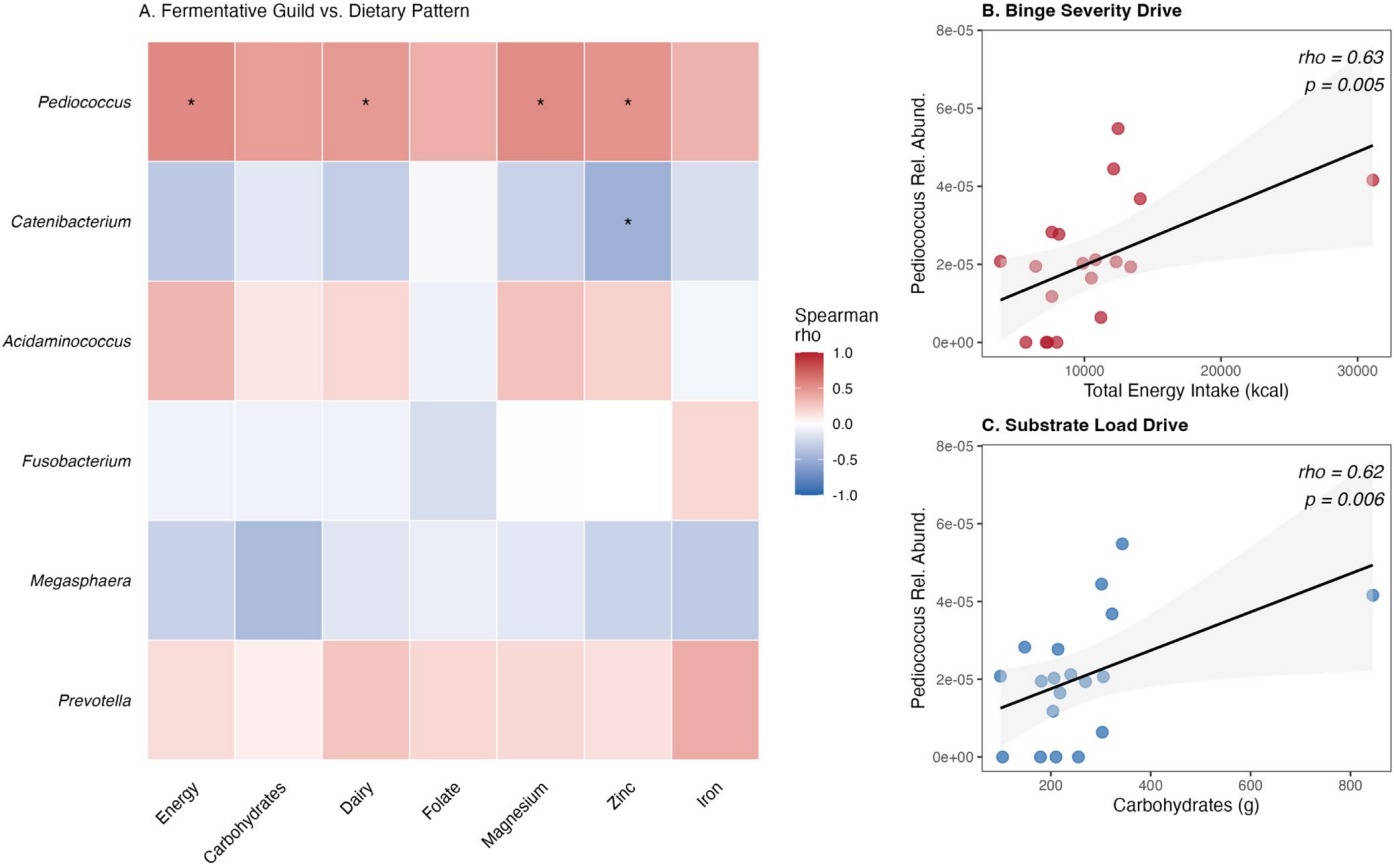
Associations between the microbial fermentative guild and dietary intake patterns in individuals with binge‐eating disorder (BED). (A) Heatmap illustrating Spearman correlation coefficients (rho) between the relative abundance of six representative genera within the fermentative guild and specific dietary variables. Significance levels are indicated by stars: *p < 0.05 . (B–C) Representative scatter plots with linear regression lines and 95% confidence intervals showing the robust positive correlations between *Pediococcus* relative abundance and (B) total daily energy intake and (C) carbohydrate intake. All analyses were performed within the BED group (*n* = 19). All correlations and *p* values presented in this figure are unadjusted; for partial correlation results adjusted for total energy intake and BDI scores, see Table 3. BDI, beck depression inventory; BED, binge‐eating disorder.

Within the BED group, initial analyses revealed robust positive associations between *Pediococcus* and multiple dietary dimensions, including total energy intake (rho = 0.63, *p*_original_ = 0.005), carbohydrates (rho = 0.62, *p*_original_ = 0.006), dairy products, and a broad profile of micronutrients (Figure 3). Notably, these associations largely remained significant when adjusting solely for BDI scores (Table 3). However, these nutritional associations lost statistical significance after adjusting for total energy intake (Table 3). This attenuation is explained by the high covariance between specific nutrients and overall caloric load within the BED group (Table S2). Other depleted genera, such as *Catenibacterium*, did not show consistent correlation patterns with dietary variables.

**TABLE 3:** Spearman correlations between *Pediococcus* relative abundance and dietary factors in the BED group.

Variable	rho_original_	FDR_original_	rho_adjusted_BDI_	*p*_adjusted_BDI_	rho_adjusted_both_	*p*_adjusted_both_
Part A: Macronutrients						
Energy (kJ)	0.63**	0.07	0.61	0.01	NA	NA
Carbohydrates (g)	0.60**	0.07	0.61	0.01	0.18	0.50
Starch (g)	0.44	0.14	0.46	0.06	0.11	0.68
Total fat (g)	0.42	0.17	0.36	0.15	−0.44	0.08
Protein (g)	0.39	0.20	0.35	0.15	−0.21	0.43
Fiber (g)	0.37	0.24	0.30	0.23	−0.01	0.97
Sugars (g)	0.32	0.30	0.33	0.18	−0.04	0.87
Part B: Food groups						
Dairy (serve)	0.50*	0.10	0.45	0.06	−0.07	0.80
Oil Equivalents (tsp)	0.40	0.20	0.38	0.12	0.15	0.58
Grains (serve)	0.23	0.50	0.28	0.26	−0.02	0.96
Vegetables (serve)	0.22	0.51	0.27	0.29	0.12	0.65
Protein Foods (serve)	0.15	0.63	0.16	0.53	−0.08	0.76
Fruit (serve)	−0.08	0.81	−0.17	0.50	−0.31	0.23
Added Sugars (tsp)	−0.02	0.96	0.02	0.94	−0.15	0.56
Alcoholic Drinks (sd)	0.01	0.96	0.09	0.72	−0.19	0.47
Part C: Micronutrients						
Folate (μg)	0.61**	0.07	0.57	0.01	0.26	0.32
Magnesium (mg)	0.58**	0.07	0.52	0.03	0.05	0.84
Calcium (mg)	0.56*	0.07	0.51	0.03	−0.12	0.64
Zinc (mg)	0.56*	0.07	0.52	0.03	0.04	0.88
Iron (mg)	0.56*	0.07	0.48	0.04	0.20	0.44
Potassium (mg)	0.53*	0.08	0.47	0.05	−0.12	0.66
Phosphorus (mg)	0.52*	0.09	0.45	0.06	−0.20	0.45
Vitamin E (mg)	0.51*	0.09	0.50	0.03	0.07	0.78
Riboflavin (mg)	0.49*	0.10	0.39	0.11	−0.15	0.57
Iodine (μg) (mg)	0.48*	0.10	0.44	0.07	−0.23	0.37
Sodium (mg)	0.35	0.25	0.36	0.14	−0.30	0.24
Vitamin C (mg)	0.31	0.33	0.41	0.10	0.24	0.36
Retinol (μg)	0.24	0.50	0.14	0.58	−0.13	0.63
Thiamin (mg)	0.22	0.51	0.19	0.46	0.06	0.83
Betacarotene (μg)	0.20	0.54	0.18	0.47	0.21	0.43
Selenium (μg)	0.17	0.62	0.13	0.60	−0.20	0.45
B12 (μg)	0.15	0.63	0.13	0.60	−0.34	0.19
B6 (mg)	0.14	0.64	0.07	0.79	−0.23	0.38
Niacin (mg)	0.07	0.84	0.04	0.87	−0.15	0.56

*Note:* Spearman correlation coefficients (rho) were calculated between the relative abundance of *Pediococcus* and dietary intake variables. rho_original_ and *p*_original_ represent the uncorrected bivariate correlation Spearman correlation coefficients. FDR_original_ denotes the false discovery rate‐adjusted *p* value calculated using the Benjamini–Hochberg method. rho_adjusted_BDI_ and *p*_adjusted_BDI_ represent partial Spearman correlations adjusted for depressive symptoms (BDI scores). rho_adjusted_both_ and *p*_adjusted_both_ represent partial Spearman correlations simultaneously adjusted for both total energy intake (Diet Energy) and BDI scores. Asterisks following the rho_original_ values indicate the significance levels: *p < 0.05, **p < 0.01.

We further examined these associations at higher taxonomic levels and in the control group to verify specificity. The strength of the correlations observed for *Pediococcus* notably diminished at the family (*Lactobacillaceae*) and order levels, indicating that these functional associations are primarily attributed to specific genera rather than broader taxonomic groups (Table S3).

In contrast, these nutritional associations were entirely absent or even reversed in the control group (Table S4). For example, *Pediococcus* showed no correlation with Folate (rho = −0.14, *p*_original_ = 0.40) and a weak negative correlation with Magnesium (rho = −0.34, *p*_original_ = 0.038) in MDD only individuals. Similarly, other depleted genera such as *Catenibacterium* and *Acidaminococcus* failed to show consistent nutritional links in either group.

### 3.5 Associations Between Gut Microbiota, Autonomic Function, and Psychological Symptoms

**FIGURE 4:**
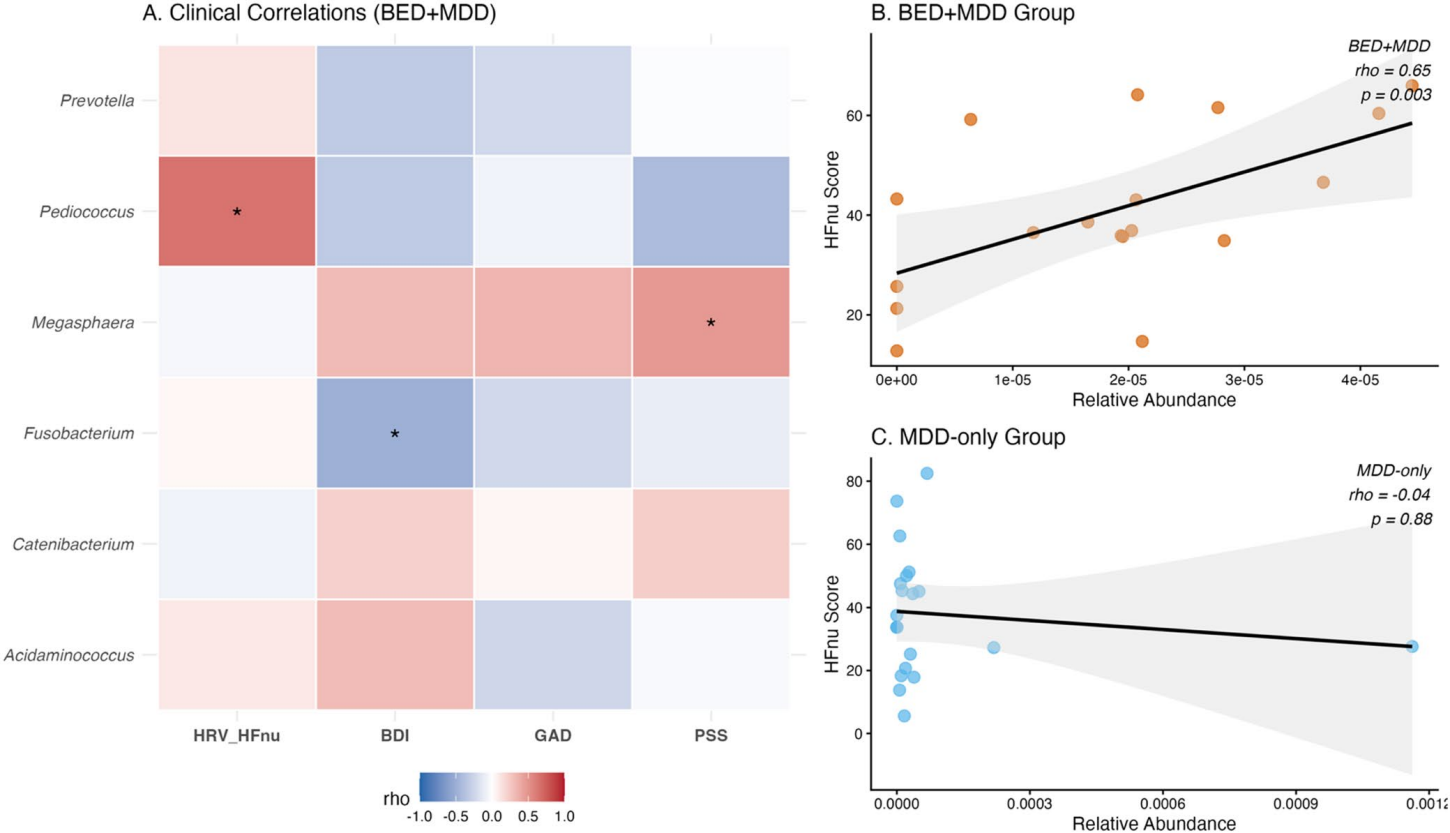
Associations between gut microbiota, autonomic function, and psychological symptoms. (A) Heatmap illustrating Spearman correlations between depleted genera and psychophysiological variables in the BED+MDD group (*n* = 19). (B, C) Scatter plots representing the association between the relative abundance of *Pediococcus* and heart rate variability (HFnu) in the BED+MDD group (*n* = 18) and the MDD‐only control group (*n* = 21), respectively. Solid lines indicate linear regression fits, and shaded areas represent 95% confidence intervals. The robust positive correlation observed in the comorbid group (rho = 0.65, *p* = 0.003) is entirely absent in the MDD‐only group (rho = −0.04, *p* = 0.88), identifying this microbial‐autonomic axis as a specific feature of the BED phenotype. BED, binge‐eating disorder; HFnu, heart rate variability high‐frequency normalized units; MDD, major depressive disorder.

Beyond nutritional intake, the depleted taxa exhibited significant correlations with autonomic and psychological profiles in the BED group (Figure 4A). The genus *Pediococcus* (prevalent in 78.95% of BED samples) was uniquely associated with cardiac autonomic regulation, showing a strong positive correlation with HRV‐HFnu (Figure 4B; rho = 0.65, *p*_original_ = 0.003). Critically, this positive correlation remained highly significant (Partial rho = 0.683, *p*_adjusted_ = 0.004) even after a stringent double‐adjustment for both BDI and total energy intake. This association was highly specific to the genus level, as it dissipated at the family and order levels (all *p* > 0.05). Also, this relationship was uniquely associated with the binge‐eating phenotype, as it completely dissipated in the MDD‐only control group (Figure 4C; rho = −0.04, *p*_original_ = 0.88).

Furthermore, we observed an inverse relationship between the *Fusobacteriales* lineage and depressive symptoms in the BED group. Specifically, both the order *Fusobacteriales* (rho = −0.66, *p*_original_ = 0.002) and the genus *Fusobacterium* (rho = −0.50, *p*_original_ = 0.031) were negatively correlated with BDI scores, suggesting that the depletion of these taxa may be linked to the depressive symptoms associated with BED. Additionally, the genus *Megasphaera* was positively associated with perceived stress (PSS: rho = 0.48, *p*_original_ = 0.039). These findings were considered in the broader interpretation of microbial–clinical relationships.

Crucially, these microbial‐clinical associations were absent in the control group (Table S5), indicating their specificity to the BED phenotype.

## 4 Discussion

### 4.1 A BED Specific Microbial Alteration: Collective Depletion of the Fermentative Guild

We identified distinct microbial differences in BED characterized by the synchronized depletion of *Pediococcus* alongside other key anaerobic fermenters, including *Fusobacterium* and *Megasphaera*. Notably, these depletions correlated with greater depressive severity and perceived stress, respectively, underscoring the psychophysiological relevance of these findings. Recognized as a probiotic with robust fermentative capacity, *Pediococcus* strains have been documented to exert beneficial effects on host metabolism, including the regulation of lipid and cholesterol levels (Damodharan et al. 2015; Wang, Xing, et al. 2025). Consequently, the specific loss of this genus may act as a sentinel for a broader functional collapse in the gut ecosystem, marking a shift away from beneficial metabolic fermentation. Within the BED group, *Pediococcus* emerged as a critical functional node, showing consistent and directionally coherent associations with a broad profile of dietary variables. Rather than being limited to isolated micronutrients, these associations formed a structured pattern that likely reflects the broader nutritional footprint of the dietary pattern and its associated substrate loads. *Pediococcus* abundance in the BED group was also linked to our HRV index (specifically HFnu, a marker of parasympathetic tone), persisting even after accounting for the overlapping effects of total caloric intake and depression severity. In contrast, the control group showed largely attenuated or flat relationships across microbiota, micronutrients and stress‐related measures. This contrast indicates that the observed association is unlikely to represent a generic feature of MDD and instead suggests a BED‐associated physiological context in which microbial and host systems vary together. However, as we did not have a direct measure of binge‐eating severity, the observed microbial associations should be interpreted as indirect correlates of the BED phenotype rather than markers of symptom intensity.

The depletion of *Pediococcus* should not be viewed in isolation but rather as a prominent manifestation of a broader ecological collapse within the fermentative guild, referring to metabolically linked bacteria that share fermentative functions. In our cohort, multiple genera belonging to the families *Erysipelotrichaceae* (e.g., *Catenibacterium*), *Acidaminococcaceae* (e.g., *Acidaminococcus*), *Lactobacillaceae* (e.g., *Pediococcus*), and *Fusobacteriaceae* (e.g., *Fusobacterium*), and genus *Prevotella*, all of which share a specialized metabolic niche by the fermentation of dietary carbohydrates and amino acids into organic acids, including lactate and short‐chain fatty acids (Jang et al. 2026; Jiang et al. 2026; Montgomery et al. 2024; Tegtmeier et al. 2016; Tett et al. 2021), exhibited synchronized reductions. This downregulation indicates systemic fermentative impairment associated with substrate volatility. While only *Pediococcus* correlates linearly with diet, it likely represents a synchronized guild depletion; with the other taxa exhibiting a floor effect (near‐zero levels), permanently suppressed within the gut environment, rather than merely fluctuating in response to daily changes in symptom severity. These results may signify a systemic collapse of the fermentative consortium in BED, rather than isolated taxonomic losses.

### 4.2 From Eating Behavior and Stress to an Altered Gut Environment

A plausible starting point for interpreting these findings is the behavioral and physiological profile that characterizes BED. BED is commonly associated with irregular eating patterns, episodes of overeating (Giel et al. 2022), and elevated chronic stress (Mathes et al. 2009), each of which can influence gastrointestinal physiology (Khalsa et al. 2022; Leigh et al. 2023). Importantly, BED is also a significant predictor of long‐term weight gain and obesity (McCuen‐Wurst et al. 2018), a state intrinsically linked to gut dysbiosis and reduced microbial diversity (Borrego‐Ruiz and Borrego 2025). Repeated overeating and irregular meal timing can alter gastric secretion dynamics and gastrointestinal load (van Dyck et al. 2021; Zipfel et al. 2006), potentially leading to fluctuations in luminal acidity (Cremonini et al. 2009) and downstream changes in gut motility (Janssen et al. 2011) and microbial habitat conditions (Schneider et al. 2024). Intestinal pH has increasingly been recognized as a key physicochemical determinant of gut microbial composition and metabolic activity, with even modest shifts capable of exerting selective pressure on microbial growth and persistence (Brinck et al. 2025). Diet‐induced changes in substrate availability and fermentation dynamics, together with host‐related physiological factors, can alter local pH conditions within the gut without requiring overt pathological acidification (Brinck et al. 2025; Flint et al. 2012). Such alterations in luminal conditions are likely to affect metabolically active gut taxa. In the present study, the BED‐specific microbial alterations were representative of a potential reduction in fermentative output, as inferred from the depletion of these functional taxa. We observed reduced abundance of key fermenters such as *Megasphaera* (associated with short‐chain fatty acid production) (Heng et al. 2025; Mutuyemungu et al. 2024; Prabhu et al. 2012), *Prevotella* (dietary fiber fermentation) (Precup and Vodnar 2019), and members of the *Fusobacteriaceae* family. These taxa thrive in stable anaerobic environments and are sensitive to the rapid pH fluctuations and osmotic loads imposed by binge‐eating episodes.

Beyond dietary patterns, the heightened affective burden in the BED group is evidenced by borderline higher BDI scores. This burden may compromise gut homeostasis through the activation of the hypothalamic–pituitary–adrenal (HPA) axis and the sympathetic‐adrenal‐medullary (SAM) system (La Torre et al. 2023; Qin et al. 2014). Activation of these stress pathways is known to impair gut barrier function and mucosal immune activity (Zhang et al. 2023). Consequently, changes in permeability and immune signaling (Ilchmann‐Diounou and Menard 2020) can modify the microbial niche and promote ecological instability observed in the BED cohort (Morys et al. 2024).

These mechanisms align with broader gut brain axis models in which behavioral stressors and dietary patterns shape the intestinal environment and thereby influence microbial community structure (Ahmed et al. 2025; Fan et al. 2023; Guo and Xiong 2024). Importantly, this conceptual framework does not require microbial changes to be the initiating event but instead positions them as an ecological response to a persistently altered gut milieu.

### 4.3 Proposed Mechanisms: Ecological Collapse Potentially Linked to Substrate Overload and Physicochemical Instability

The continuous changes in gut pH that accompany BED exert a profound selective pressure on the microbiome. The intestinal microbial balance is fragile and highly dependent on a stable pH environment within narrow limits. An imbalanced pH disrupts this ecosystem by promoting the overgrowth of harmful pathobionts while simultaneously suppressing beneficial, symbiotic microbes such as the butyrate‐producing *Fusobacterium* identified in our study, a major producer of SCFAs like butyrate. This reduction in butyrate increases the risk of elevated gut permeability, leading to consequent proinflammatory responses that are known promoters of mood disorders and end‐organ metabolic dysfunction (Firrman et al. 2022).

Within an altered gut ecosystem, microbial taxa differ in their capacity to resist physicochemical fluctuations (Fassarella et al. 2021). In the context of BED, binge‐eating episodes are frequently characterized by the rapid consumption of highly palatable, carbohydrate‐rich foods (Moraes et al. 2023). This massive influx of rapidly fermentable substrates can overwhelm the carrying capacity of the proximal colon, leading to runaway fermentation and precipitous drops in luminal pH (Flint et al. 2012).

While *Pediococcus* is inherently an acid‐tolerant lactate producer, its persistence relies on a stable ecological network, particularly the presence of lactate‐utilizing bacteria such as *Megasphaera* that prevent lactate accumulation (Belenguer et al. 2007). Our results showed a synchronous depletion of *Megasphaera* alongside *Pediococcus*. *Megasphaera* is notoriously sensitive to rapid acidification (pH < 5.5) (Walker Alan et al. 2005). We propose that the rapid acidification induced by binge‐eating may first eliminate these crucial lactate‐consumers. The consequent accumulation of unbuffered lactate creates a toxic environment of end‐product inhibition, where even acid‐producing taxa like *Pediococcus* cannot sustain metabolic activity or growth (Wang, Gong, et al. 2025).

Furthermore, the sheer volume of food consumed during binges imposes acute osmotic stress. Experimental evidence indicates that under conditions of sustained acid stress, characterized by reduced environmental pH and inflammation‐related metabolic burden, *Pediococcus* exhibits impaired functional stability, with diminished metabolic activity and reduced capacity to persist within the microbial community (Wang, Gong, et al. 2025). Evidence from controlled physicochemical stress experiments further supports this interpretation. In a non‐host system where pH was experimentally reduced, several fermentative taxa, including *Prevotella*, *Megasphaera*, and *Acidaminococcus*, exhibited marked ecological collapse, mirroring the taxa significantly reduced in the BED group and suggesting that acid‐related environmental stress may contribute to their loss (Zhang et al. 2019). Therefore, the BED gut likely represents a hostile niche characterized by chemical volatility. In this unstable environment, taxa requiring cooperative cross‐feeding (like the *Pediococcus*‐*Megasphaera* axis) are particularly vulnerable to ecological collapse, distinct from the stable suppression seen in chronic conditions like depression.

### 4.4 Emergence of Cross System Association: Microbiota, Stress Physiology and Dietary Patterns

The most distinctive feature of the BED group was the emergence of cross system correlations, where *Pediococcus* was associated with both nutritional intake and HRV. Specifically, *Pediococcus* abundance showed robust positive correlations with the intake of carbohydrates and dairy products, representing key fermentable substrates, as well as essential micronutrients. The micronutrients associated with *Pediococcus* were not arbitrary but included nutrients that are central to metabolic regulation and neurobiological function, including folate and minerals (zinc, magnesium) that serve as essential enzymatic cofactors for neurotransmitter synthesis (Chamberlain et al. 2018; Sarris et al. 2015). Such micronutrients are routinely implicated in pathways relevant to one‐carbon metabolism, which are processes relevant to biosynthesis, homeostasis, epigenetic maintenance, and redox defense (Ducker and Rabinowitz 2017; Friso et al. 2017). The unique presence of these associations in the BED group, but not in MDD controls, identifies a specific metabolic pattern where microbial stability is intrinsically tied to the nutritional landscape.

There are likely two explanations for why these dietary associations lost statistical independence after adjusting for total energy. These findings may reflect either nutrient‐specific microbial relationships or the broader metabolic impact of fluctuating energy intake associated with binge‐restrict cycles. The strong correlation between specific nutrients and total energy suggests that these findings reflect the impact of overall binge‐eating patterns. The massive influx of food and the resulting changes in gut acidity during binge‐eating episodes likely create an environment where beneficial bacteria struggle to survive. Importantly, given the modest sample size (*n* = 19), specific nutrient effects may have been statistically masked by high shared variance with overall caloric load. Larger cohort studies are required to determine whether these micronutrients possess unique physiological roles beyond their association with binge‐related caloric surges.

At the same time, the association between *Pediococcus* and HRV index suggests a direct pathway of microbial‐neural communication, likely mediated via the vagus nerve. HFnu is a widely used indicator of autonomic balance and vagal tone (Laborde et al. 2017; Pham et al. 2021). The *Lactobacillaceae* family, to which *Pediococcus* belongs, is well documented for its capacity to stimulate the vagus nerve, thereby enhancing parasympathetic activity and reducing physiological stress reactivity (Bravo et al. 2011).

However, a mechanistic explanation for this association extends beyond simple correlation. Generally, *Pediococcus* species are not permanent residents but transient commensals originating from fermented foods (Fischer et al. 2025). We propose that their specific depletion in BED is related to the rapid consumption rates and substrate overload characteristic of binge‐eating episodes, which create chronic intestinal pH instability. This volatile environment likely prevents the colonization of these transient beneficial microbes, instead favoring the proliferation of pathobionts.

**FIGURE 5:**
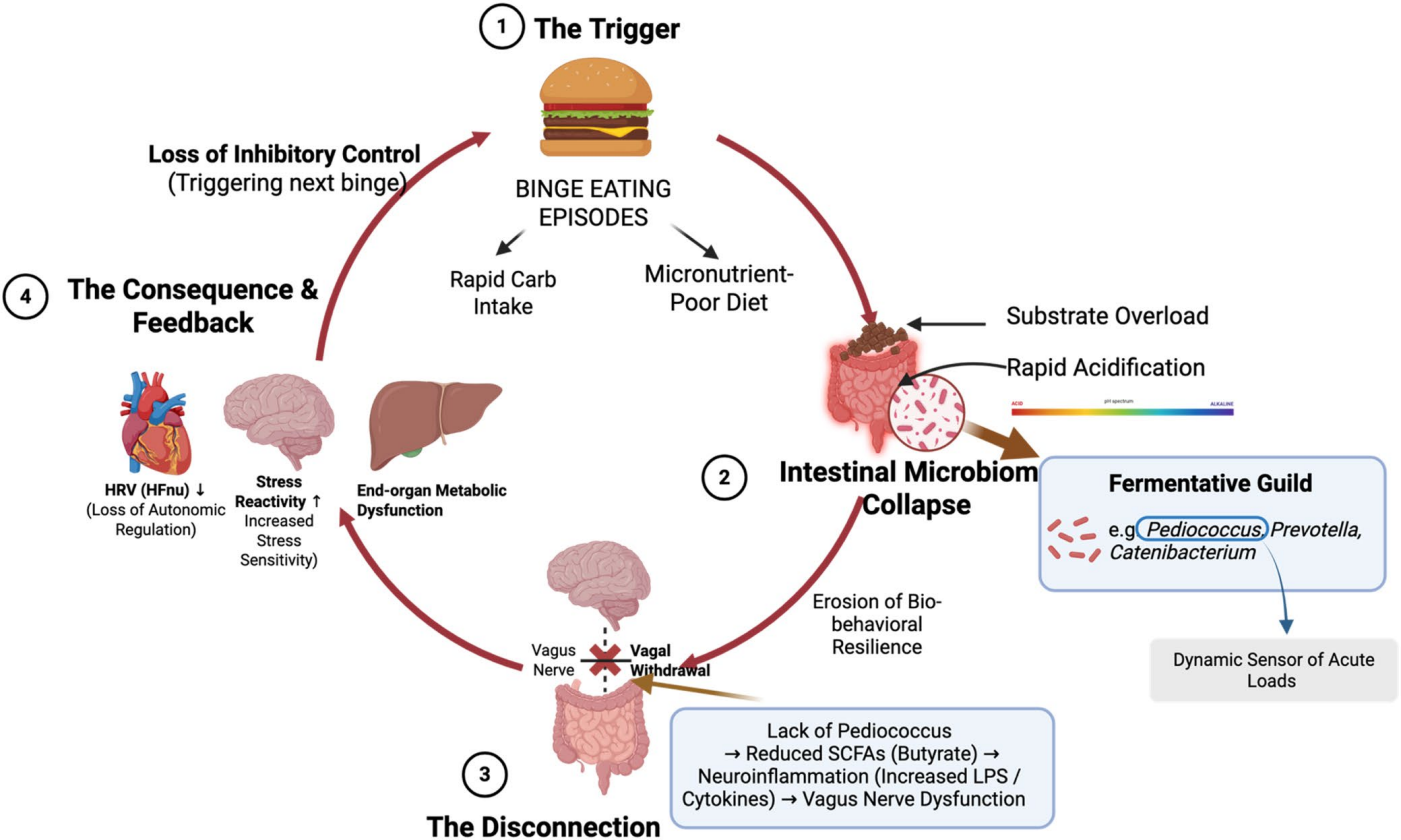
Proposed “bio‐behavioral feedback loop” mechanism in binge‐eating disorder (BED). This schematic presents a proposed biobehavioral framework based on the associations observed in the current data. (1) The Trigger: Binge‐ eating episodes introduce massive carbohydrate loads, osmotic stress, and rapid consumption rates. (2) The Intestinal Microbiome Collapse: Substrate overload is proposed to associate with rapid luminal acidification and pH instability. This shifting environment may impact the presence of fermenters. (3) The Disconnection: The systemic depletion of these taxa results in a critical reduction of Short‐Chain Fatty Acids (SCFAs). This metabolic deficit is proposed to potentially promote neuroinflammation, contributing to vagus nerve tone dysfunction and diminished parasympathetic signals (low HRV‐HFnu). (4) The Consequence & Feedback: The chronic gut dysbiosis is proposed to relate to end‐organ metabolic dysfunction and impaired autonomic regulation. These physiological stressors exacerbate stress reactivity and reduce inhibitory control, thereby triggering further binge episodes and perpetuating the potential feedback loop.

In our BED cohort, the depletion of *Pediococcus* was strongly correlated with reduced HRV, implying a dampening of this microbial vagal support. This impairment is likely compounded by the broader loss of fermentative capacity described above. SCFAs, particularly butyrate, serve as a vital activator and energy source for the gut‐brain neural circuits, directly linking microbial metabolism to vagal tone (De Vadder et al. 2014). Consequently, the reduced availability of these metabolites may negate the potential probiotic actions of *Pediococcus*, effectively dismantling the microbial support system necessary for mitigating gut dysbiosis and preserving heart health. Beyond being a passive responder to stress‐related physiological changes, *Pediococcus* itself has been shown to exert direct stress‐modulating effects. Experimental and clinical studies demonstrate that *Pediococcus acidilactici* supplementation can alleviate depressive and stress‐related profile, reduce neuroinflammation, and improve autonomic and behavioral outcomes, highlighting its role as a psychobiotic (Li et al. 2025; Sun et al. 2025). This ecological shift has profound physiological consequences. The loss of *Pediococcus* and the concurrent rise in pathobionts can lead to increased systemic lipopolysaccharide (LPS) translocation. Elevated LPS is a known driver of neuroinflammation and HPA axis dysregulation (Kelly et al. 2015), which directly suppresses vagal tone. The systematic collapse of the fermentative guild further exacerbates this state by stripping the host of its microbial metabolic buffer. Thus, the association observed here represents a bio‐behavioral cycle: aberrant feeding patterns create a hostile gut environment that dismantles microbial vagal support, thereby potentially contributing to a physiological environment that mirrors the persistence of the disorder (Figure 5).

### 4.5 Why This Coupled State Appears in BED but Not in Depression Alone

The contrast between BED plus MDD and MDD alone suggests that depression alone is not sufficient to generate the observed associations, and that BED introduces an additional context. At a behavioral level, this may simply reflect the inherent instability of eating patterns in BED, where concentrated binge‐eating episodes impose acute substrate shocks that likely disrupt the metabolic utilization window for micronutrients. Such a disrupted window provides a plausible basis for these differences, potentially reflecting reduced efficiency in micronutrient utilization, specific metabolic sensitivity to nutrient fluctuations, or both.

Although estimated dietary intake appeared comparable between groups, BED is commonly accompanied by alterations in gastrointestinal physiology (Alyami et al. 2023; Geliebter et al. 2004; Peat et al. 2013). Moreover, even when mean intake is similar, BED is characterized by episodes of concentrated intake over short time windows (Guha 2014), with a large proportion of daily energy consumed during discrete binge events (Moraes et al. 2023). This pattern may impose acute gastrointestinal and metabolic loads that differ from more evenly distributed eating patterns (Alagha et al. 2025; Parry et al. 2017), potentially limiting the efficiency with which micronutrients are absorbed and utilized across the day (Schultz et al. 2025). Although total energy intake was similar across both groups, the significant correlation with microbial abundance was exclusive to the BED cohort. This finding suggests that the relationship is not merely a result of the total amount of food consumed. Instead, it likely reflects a unique sensitivity of the BED microbiome to caloric intake or the specific impact of irregular eating patterns. As a result, metabolic pathways requiring relatively continuous micronutrient support may be particularly sensitive to this instability despite comparable average intake. Beyond host absorption processes, gut microbiota itself can contribute to the effective availability of micronutrients. Several taxa within the *Lactobacillaceae* family have been linked to the bioaccessibility and utilization of multiple minerals, primarily through fermentation‐related processes that modify the intestinal chemical environment (Bielik and Kolisek 2021). In addition, lactic acid bacteria (LAB), such as *Pediococcus*, are capable of synthesizing B‐group vitamins including folate (Mahara et al. 2023), indicating that microbial metabolism may represent an additional, diet‐independent source of micronutrients within the gut ecosystem.

In parallel, histamine‐related pathways may contribute to increased downstream metabolic demand in BED. Histamine degradation via histamine N‐methyltransferase requires S‐adenosylmethionine, directly drawing on one‐carbon metabolism (Moya‐García et al. 2021). Evidence indicates that intestinal histamine is not derived predominantly from dietary sources but arises from the combined contribution of host cells and gut microbiota (Smolinska et al. 2022). Microbially derived histamine, produced by histidine decarboxylation in a range of bacterial taxa, may therefore constitute a persistent background metabolic load within the gut environment (Smolinska et al. 2022). Importantly, bacterial histidine decarboxylase activity is upregulated under acidic conditions as part of an adaptive response to physicochemical stress, linking altered gut environments to increased histamine production (Smolinska et al. 2022). Increased histamine turnover could consequently elevate demand for methyl donors without changes in dietary intake, providing a plausible basis for increased one‐carbon metabolic demand.

Under this interpretation, depression alone appears to primarily involve alterations in central stress and affective regulation, while downstream gastrointestinal physiology may remain comparatively buffered (Bhatia and Tandon 2005). In contrast, BED is characterized by the convergence of multiple layers of physiological challenge, including repeated binge‐eating behaviors, sustained psychological stress, and a higher prevalence of functional gastrointestinal disorders reported in eating disorder populations, including BED (Santonicola et al. 2019). Together with well‐described alterations in gastrointestinal motility and autonomic regulation associated with a binge‐type profile (Zipfel et al. 2006), these factors are likely to render the gastrointestinal environment more sensitive to perturbation and less resilient to repeated stimulation. Rather than reflecting a single BED‐specific pathological mechanism, the coupled patterns observed in BED may therefore arise from a state of heightened physiological vulnerability, in which gut‐related processes, metabolic pathways, and stress‐responsive systems are more likely to interact. Unlike MDD, which is primarily characterized by central affective dysregulation and relatively stable dietary habits, BED involves a unique convergence of behavioral instability and metabolic dysregulation (insulin/glucose spikes from binges) and autonomic instability (sympathetic dominance during stress/binge cycles). This specific physiological milieu may render the gut‐brain axis more susceptible to the locking of microbial and host rhythms observed in our study.

### 4.6 Strengths, Limitations and Future Directions

A major strength of this study is its integrative and exploratory perspective. By jointly examining gut microbiota, dietary micronutrient intake, psychological measures and physiological stress markers, this work moves beyond single domain approaches that dominate much of the eating disorder literature. This multi‐system framework is particularly suited to BED, a condition characterized by complex interactions between behavior, physiology and metabolism, and allows the identification of coordinated patterns that may otherwise remain undetected. While this integrative framework offers a novel perspective on BED pathophysiology, this model should be considered hypothesis generating and requires validation in longitudinal and experimental designs to confirm the temporal sequence of these associations.

Several limitations should be acknowledged. The sample size was modest, particularly for the BED group, and the findings should therefore be interpreted as hypothesis generating rather than definitive. In addition, the cross‐sectional design precludes causal inference or the ruling out of bidirectional effects. A primary limitation is the absence of direct measures for binge‐eating frequency or severity (e.g., EDE‐Q). As we did not have a direct measure of binge‐eating severity, the observed microbial associations should be interpreted as indirect correlates of the BED phenotype rather than markers of symptom intensity. Furthermore, our focus on a comorbid BED and MDD cohort provides insight into a prevalent subgroup but may not generalize to the broader BED population without clinical depression. While we adjusted for depressive symptoms via covariance analysis, the higher baseline BDI scores in the BED group suggest that future prospective studies should employ more stringent matching criteria. Moreover, our proposed physiological mechanisms remain hypothetical due to the lack of direct functional measures, such as fecal SCFA concentrations, gastric acidity, or biological indicators of micronutrient status. Lastly, it must be acknowledged that data regarding race and ethnicity were unavailable in the source database, which restricts our ability to generalize the findings across diverse ethnic and racial groups and to examine potential demographic‐specific variations in the observed results.

Future studies should aim to replicate these findings in larger cohorts and incorporate longitudinal designs. Importantly, including direct measures of binge‐eating frequency and severity would allow for a more granular analysis of how specific behaviors trigger microbial shifts. The inclusion of biomarkers related to micronutrient status, autonomic regulation, inflammatory activity, and histamine‐related pathways would enable more direct testing of the proposed mechanisms and their relevance for targeted interventions.

## 5 Conclusion

In conclusion, this study identifies a distinct bio‐behavioral profile in BED, characterized by pathological associations between dietary pattern, gut microbiota, and autonomic physiology. Unlike the disconnect observed in depression alone, BED appears to involve a self‐reinforcing potential feedback loop: dietary instability relates to the depletion of metabolically specialized taxa (e.g., *Pediococcus*), which in turn is linked to diminished microbial support for vagal tone, associating with a state of heightened stress reactivity in individuals with BED. These findings challenge the traditional view of BED as a purely behavioral disorder, highlighting the gut microbiome as a crucial, yet overlooked, mediator of physiological dysregulation. While these cross‐sectional associations require validation in larger, longitudinal cohorts, they offer a conceptual framework for understanding the somatic underpinnings of binge‐eating. Future research should prioritize mechanistic investigations to determine whether restoring this gut‐brain link could eventually serve as a tangible target for precision interventions, moving beyond a purely psychological understanding of the disorder.

## Author Contributions

**Shuang Liang:** conceptualization, formal analysis, investigation, visualization, writing – original draft, writing – review and editing, validation, methodology, data curation. **Esben Strodl:** conceptualization, methodology, supervision, writing – review and editing, validation, data curation. **Leanne Barron:** supervision, writing – review and editing. **Matthew Bambling:** writing – review and editing, conceptualization, methodology. **Luis Vitetta:** conceptualization, writing – review and editing, methodology.

## Lived Experience Involvement Statement

No specific efforts were undertaken to involve persons with lived experience in the study design or execution, or in the preparation of this manuscript.

## Funding

This secondary analysis received no specific funding. The parent randomized controlled trial was funded by a grant from Medlab Clinical.

## Ethics Statement

The parent trial received human research ethics approval from the University of Queensland Human Research Ethics Committee and the Queensland University of Technology Human Research Ethics Committee. The trial was prospectively registered with the Australian and New Zealand Clinical Trials Registry (ACTRN12617000419369) and conducted in accordance with CONSORT guidelines.

## Conflicts of Interest

The parent trial was funded by a grant from Medlab Clinical. L.V. was a previous employee of Medlab Clinical. The other authors declare no conflicts of interest.

## Supporting information


**Table S1:** Differential abundance analysis results at multiple taxonomic levels. (A) Order level; (B) Family level; (C) Genus level. All results were generated using DESeq2 comparing BED+MDD vs. MDD.
**Table S1A:** Order level results.
**Table S1B:** Family level results.
**Table S1C:** Genus level results.

**Table S2:** Correlation between Specific Nutrients and Overall Caloric Load within the BED Group.

**Table S3:** Correlation analysis between micronutrients and microbial abundance at higher taxonomic levels in the BED+MDD group. Results are presented for (A) Order level and (B) Family level.
**Table S3A:** Spearman correlation coefficients between micronutrients and the order level in the BED+MDD group.
**Table S3B:** Spearman correlation coefficients between micronutrients and the family level in the BED+MDD group.

**Table S4:** Correlation analysis between micronutrients and microbial abundance at three taxonomic levels in the healthy control group. Associations were examined at the (A) Order level, (B) Family level, and (C) Genus level within the MDD.
**Table S4A:** Correlation analysis results at the Order level in MDD.
**Table S4B:** Correlation analysis results at the Family level in MDD.
**Table S4C:** Correlation analysis results at the Genus level in MDD.

**Table S5:** Associations between identified microbial genera, psychological symptoms (A), and autonomic function (B) in the MDD group.
**Table S5A:** Correlation between the relative abundance of identified genera and psychological symptoms in the MDD group.
**Table S5B:** Correlation between the relative abundance of identified genera and heart rate variability (HFnu) in the MDD group.

## Data Availability

Data from the parent randomized controlled trial are not publicly available due to ethics and privacy restrictions but may be obtained from the corresponding author upon reasonable request and institutional ethics approval.
